# Supplementary material for: Structure of the MlaC-MlaD complex reveals molecular basis of periplasmic phospholipid transport
Source: Nat Commun. 2024 Jul 30;15:6394. doi: 10.1038/s41467-024-50615-3 (PMC11289387; doi:10.1038/s41467-024-50615-3)
Supplement: Supplementary file 1 — Supplementary Information [file 41467_2024_50615_MOESM1_ESM.pdf]

Supplementary Information

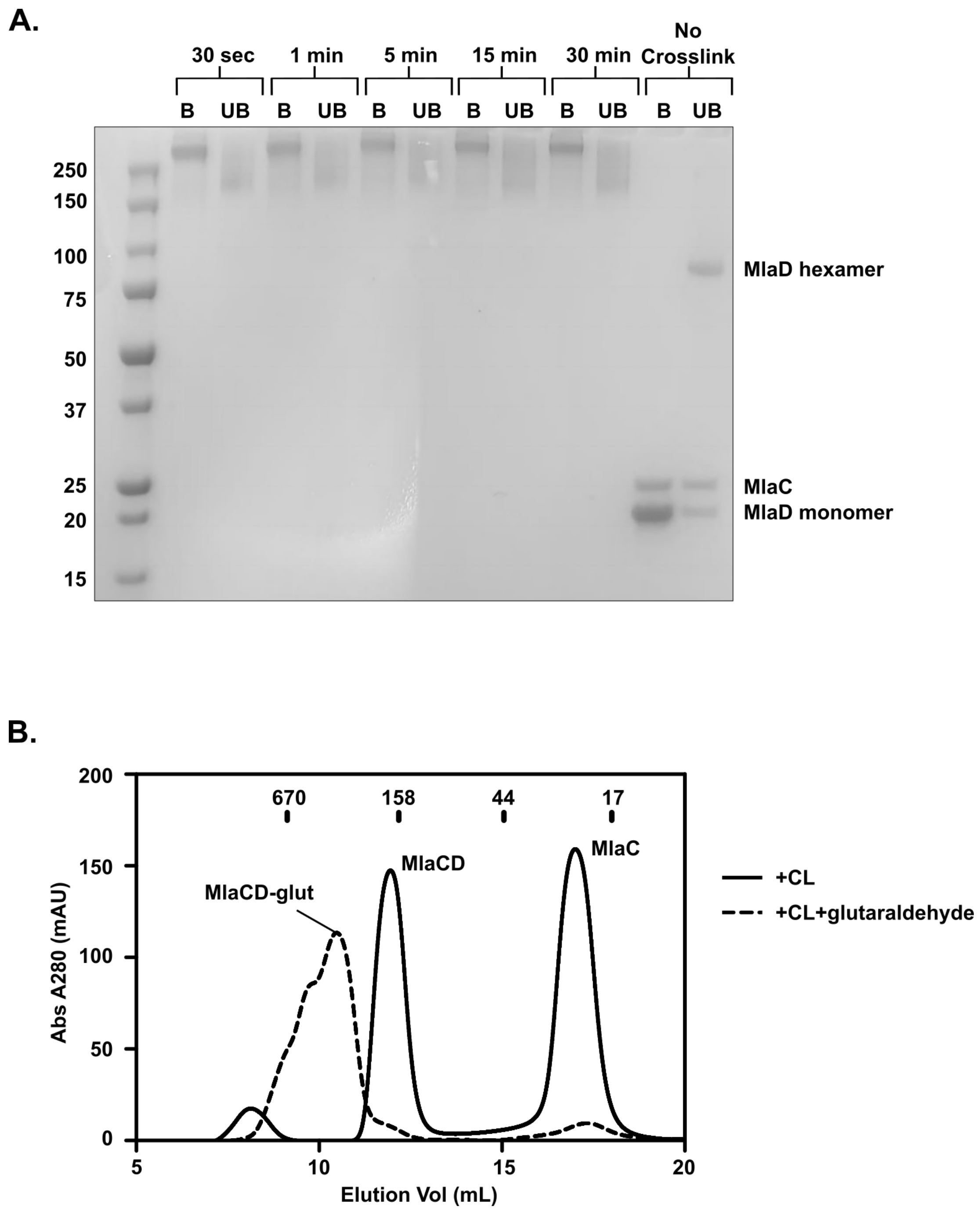

Supplementary Figure 1 – Crosslinking of the MlaCD complex for cryo-EM analysis

A) SDS-PAGE of the effect of glutaraldehyde exposure on MlaCD complex stabilisation under boiled (B) and unboiled (UB) conditions **B)**. Size exclusion chromatogram (Superdex 200) showing the elution volume between MlaCD pre and post glutaraldehyde cross-linking. Source data are provided as a Source Data file.

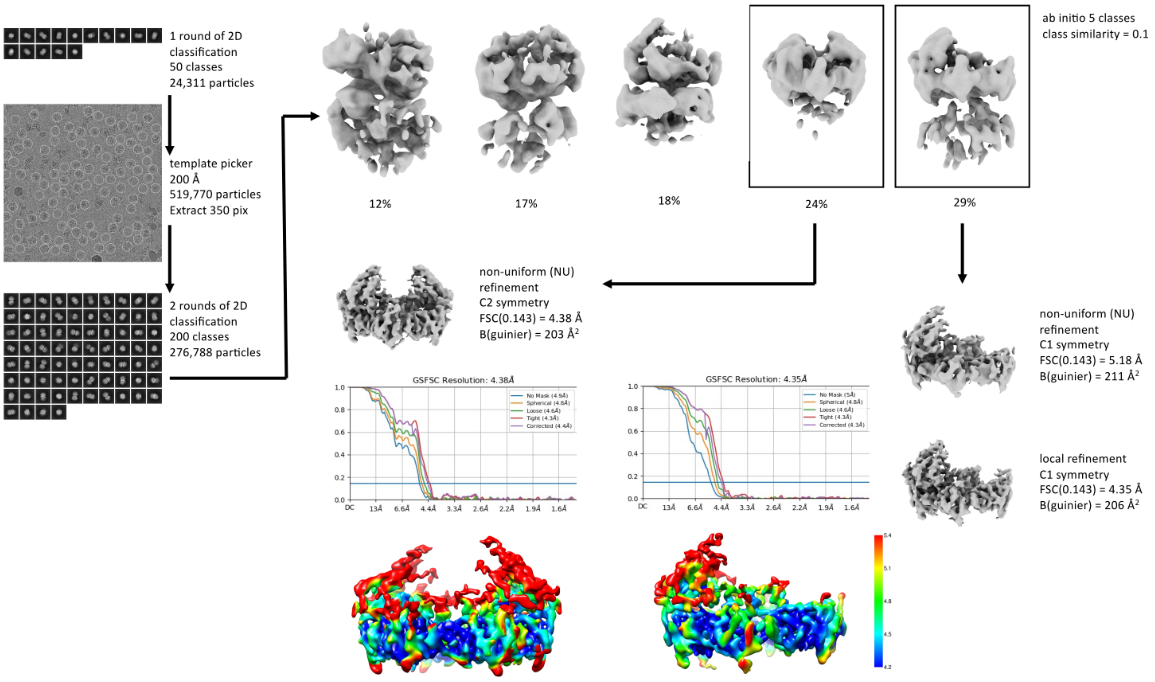

Supplementary Figure 2 – Processing pipeline of the MlaCD dataset in cryoSPARC v3.3.1. A stack of 24,311 particles were used to generate 2D class averages. The best classes were used to re-pick particles template-based. After two rounds of 2D classification multi class *ab initio* 3D maps were generated. The two best looking maps corresponding to maps with one or two molecules of MlaC bound were refined using cryoSPARCs Non-Uniform (NU) refinement procedure with C1 and C2 symmetry, respectively, followed by local refinement of the C1 map. The final particle stack for the C1 map contained 97,460 particles and was local refined to 4.35 Å (FSC=0.143). The final particle stack for the C2 map was 58,259 and NU refined to 4.38 Å (FSC=0.143). Fourier Shell Correlation plots are shown as well as local resolutions at FSC=0.5 projected on the final maps. A histogram with the full local resolution range (from 4.2 Å in blue to 5.4 Å in red) is also indicated.

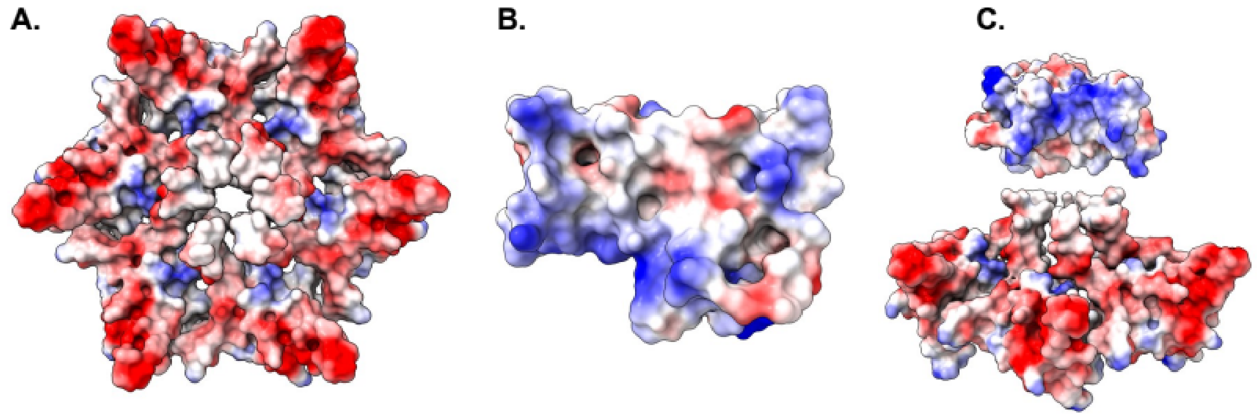

Supplementary Figure 3 – Electrostatic surface representations of MlaD. A) Electrostatic surface representation of the periplasmic face of the MlaD hexamer. The surface is largely negatively charged (red) with hydrophobic patches concentrated in central pore region, and noticeable positively charged (blue) patch at the base of the C-terminal helices. **B)** Electrostatic surface representation of MlaC displaying the surface which interacts with MlaD. The surface has an overall positive charge particularly near the edges with hydrophobic and negatively charged patches localized towards the middle. **C)** Electrostatic surface representation of the binding interfaces of MlaC and MlaD displaying their charge complementarity.

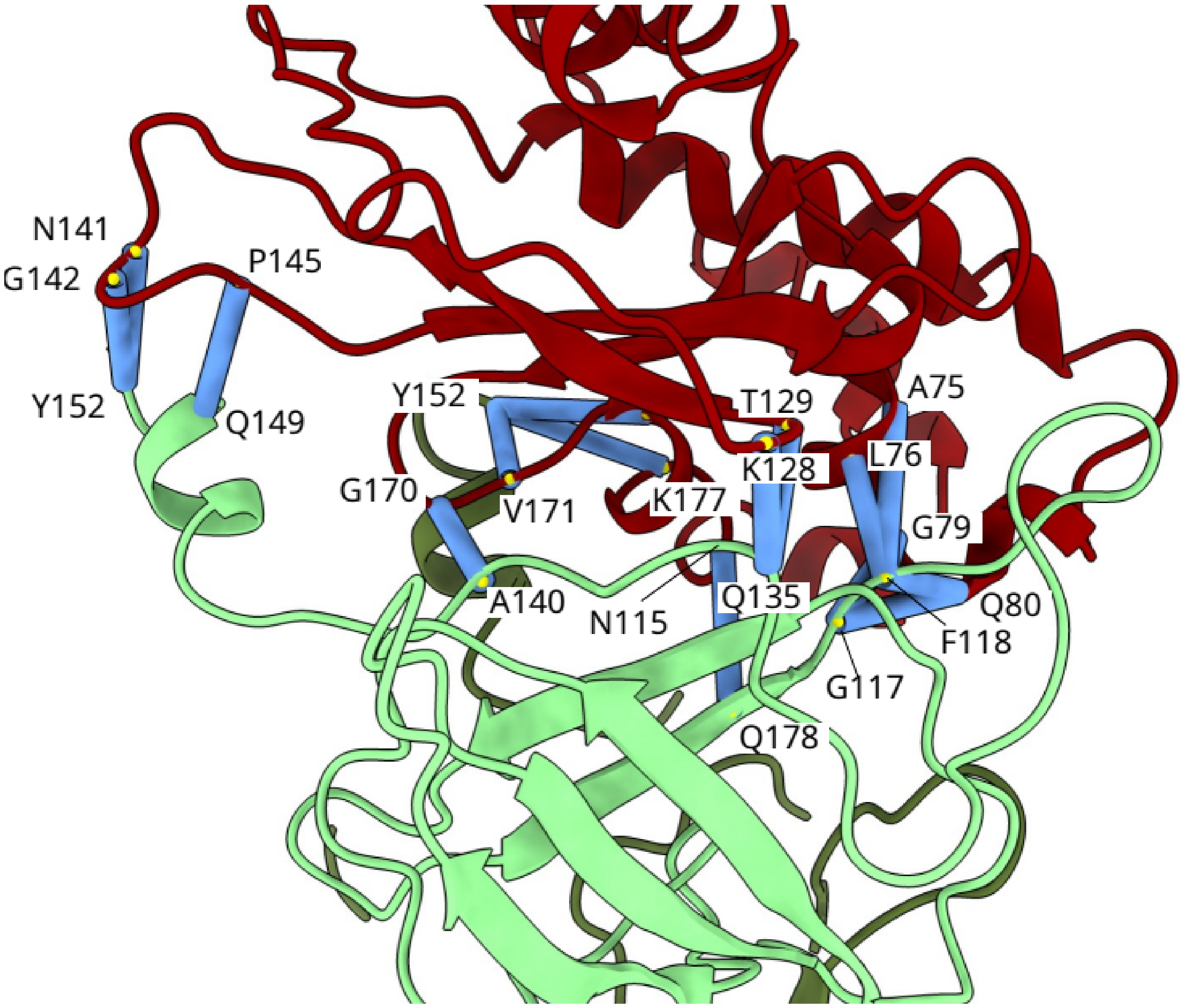

Supplementary Figure 4 – The binding interface between MlaC and MlaD is shown, with the proteins coloured in red and green, respectively. Residues determined to have high co-evolution correlation (> 0.4, see table S2) using RaptorX (Zeng et al. 2018) are highlighted with a blue line.

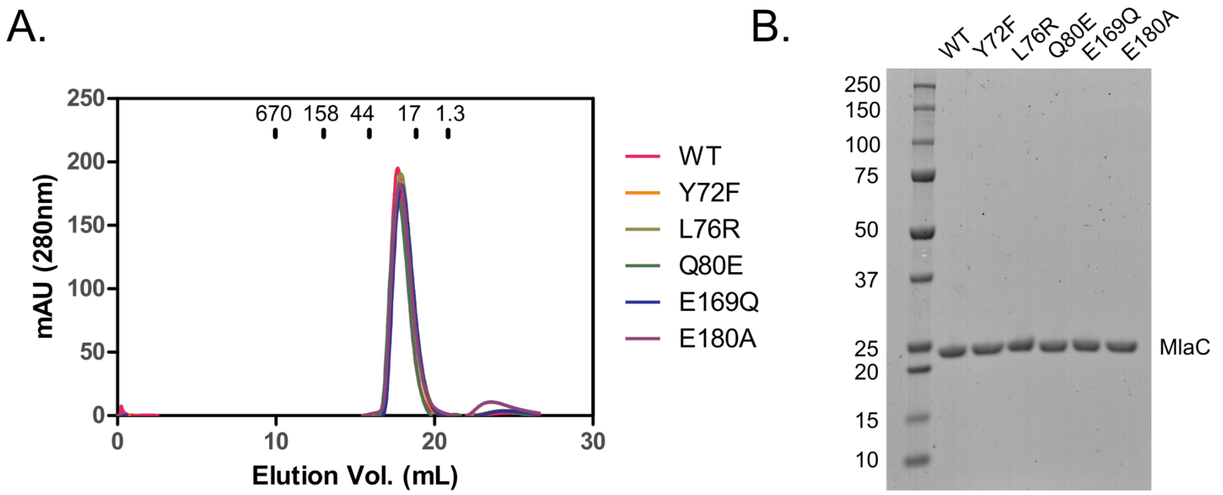

Supplementary Figure 5 – Size exclusion chromatography traces and SDS-PAGE of purified MlaC mutants used in this study. A) SEC traces of purified MlaC mutants B) SDS-PAGE of MlaC mutants following purification, samples were not boiled before electrophoresis. Source data are provided as a Source Data file.

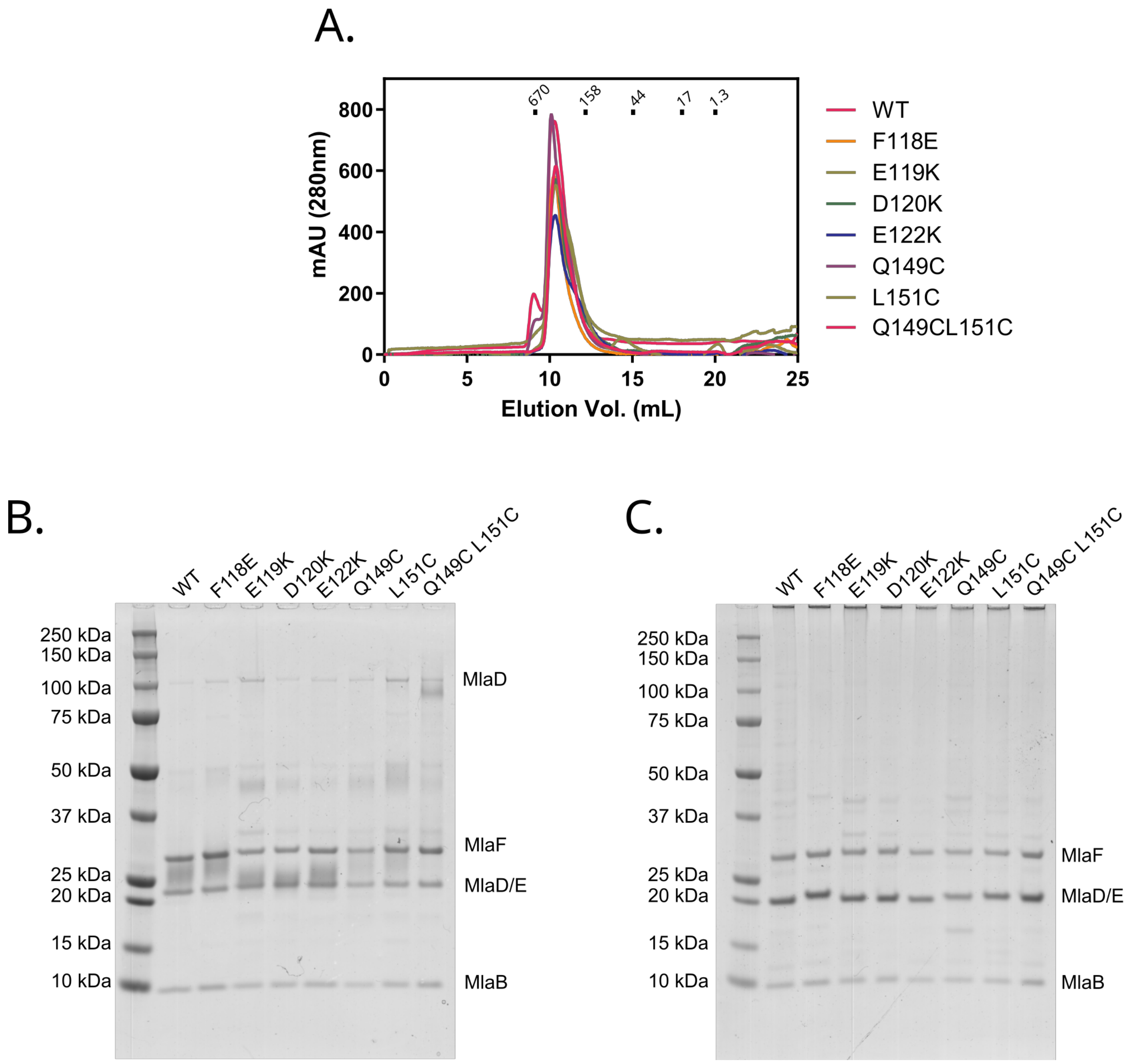

Supplementary Figure 6 – Size exclusion chromatography traces and SDS-PAGE of purified MlaFEDB mutants used in this study. A) SEC traces of purified MlaFEDB mutants B) MlaFEDB mutants, samples were not boiled before electrophoresis and bands associated with MlaD can be seen at various stages of unfolding C) MlaFEDB mutants, samples were boiled at 90 °C for 5 min before electrophoresis. Source data are provided as a Source Data file.

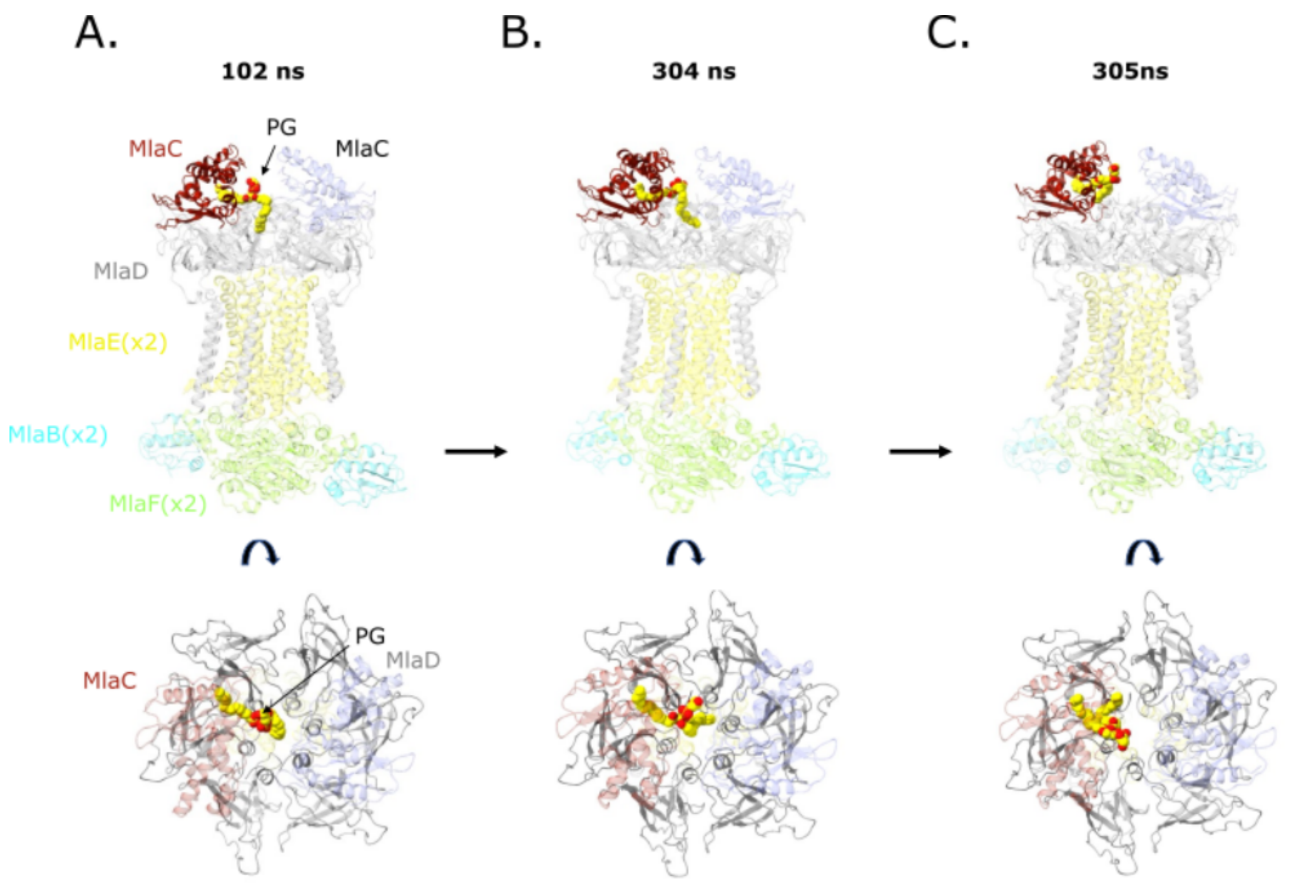

Supplementary Figure 7 – Spontaneous binding of free PG to MlaC in MlaFEDB-MlaC membrane-bound complex during 5µs coarse-grained MD simulation. **A)** Frame at 102 ns of simulation showing PG simultaneously bound to MlaD (grey) and MlaC (maroon) after initial free lipid binding. **B)** Frame at 304 ns showing PG remains bound to both MlaC and MlaD. **C)** Frame at 305 ns as the bound PG spontaneously binds to solely MlaC. Side view (top panel) and top view (bottom panel) representations of complex converted from CG to AT via CG2AT. MlaFEB structure from PDB entry 7CGE and MlaD helices modelled using AlphaFold.

A.

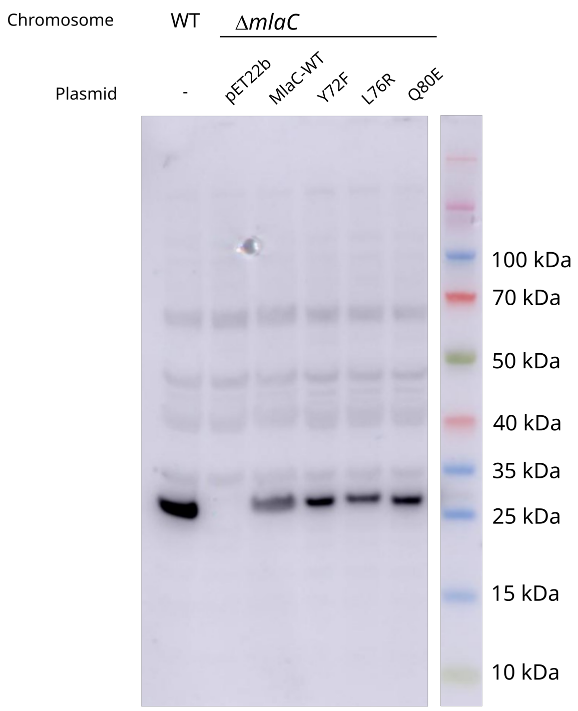

B.

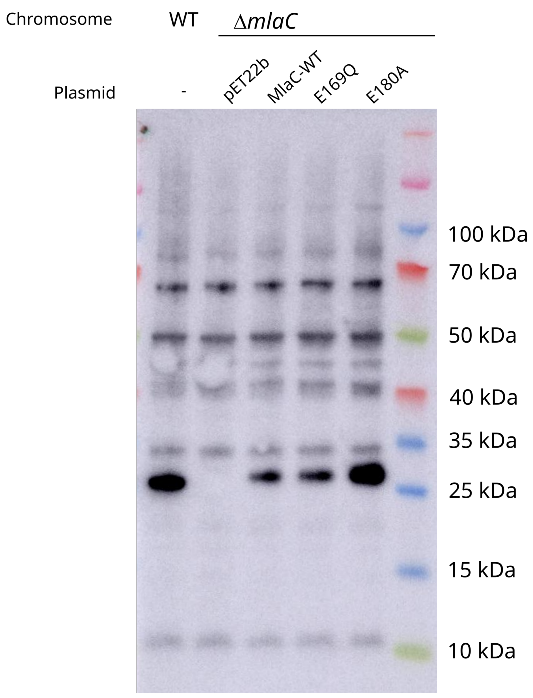

Supplementary Figure 8 – Raw images corresponding to Figures 5 & 6. A) Complete image of the western blot corresponding to Figure 5A showing relative expression levels of MlaC in ΔmlaC cells supplemented with various plasmids compared against the WT. **B)** Complete image of the western blot corresponding to Figure 6D showing relative expression levels of MlaC in ΔmlaC cells supplemented with various plasmids compared against the WT. Source data are provided as a Source Data file.

Supplementary Table 1 – CryoEM refinement statistics

**Data collection**		
Voltage (kV)	300	
Exposure (e/Å2)	40	
Fractions	50	
Defocus range ([illegible]m)	-0.5 to -2	
Pixel size (Å pix-1)	0.75	
Number of micrographs	8,741	
Initial particle number	519,770	
**Map refinement**		
Final particle number	97,460	58,259
Resolution (Å)	4.35	4.38
Symmetry	C1	C2
EMDB entry	EMD-16904	EMD-16913
**Structure refinement**		
Non-hydrogen atoms	6,983	8,348
Protein residues	894	1,065
Protein B-factor	75.39	40.21
Bond length RMSD (Å)	0.004	0.003
Bond angle RMSD (º)	0.808	0.737
MolProbity score	2.1	2.23
Clash score	7.71	9.46
Poor rotamers	0.26	0.22
Ramachandran favoured (%)	83.86	80.46
Ramachandran allowed (%)	15.91	19.45
Ramachandran disallowed (%)	0.23	0.1
PDB entry:	8OJ4	8OJG

Supplementary Table 2 – Top 50 residues pairs determined to have high co-evolution correlation determined using RaptorX (Zeng et al. 2018)

MlaC Residue No.	MlaD Residue No.	Co-evolution Likelihood
75	118	0.744819
79	117	0.687065
79	118	0.65354
76	118	0.615
80	118	0.599091
80	117	0.55884
75	117	0.55482
75	119	0.518157
79	116	0.515006
76	136	0.484357
76	117	0.479572
81	117	0.469245
78	117	0.465682
81	116	0.460129
80	116	0.43466
129	135	0.433084
157	121	0.431463
157	122	0.420946

130	135	0.4079
186	79	0.398256
84	76	0.38232
83	120	0.379392
78	118	0.373629
80	119	0.359825
83	123	0.357175
72	118	0.354696
75	120	0.353026
80	75	0.345905
169	152	0.342538
81	118	0.336381
80	120	0.328156
112	28	0.322782
83	127	0.320571
83	119	0.317352
87	123	0.317239
178	96	0.317149
72	119	0.315136

84	75	0.314543
108	28	0.31365
81	127	0.305265
28	6	0.301419
170	152	0.294484
128	135	0.293041
78	116	0.291384
83	118	0.290757
207	96	0.29067
27	107	0.286999
176	148	0.284189
156	121	0.281259
187	76	0.280711

Supplementary Table 3 – Bacterial strains used in this study

Strains	Relevant genotypes and characteristics	Reference
BL21(DE3)	fhuA2 [lon] ompT gal (λ DE3) [dcm] ΔhsdS<br>λ DE3 = λ sBamHIo ΔEcoRI-B int::(lacI::PlacUV5::T7 gene1) i21 Δnin5	NEB
BW25113	F- LAM- rrnB3 DElacZ4787 hsdR514 DE(araBAD)567 DE(rhaBAD)568 rph-1	CGSC
JW3160	BW25113 ΔmlaD (Keio library)	Baba 2006
JW3159	BW25113 ΔmlaD (Keio library)	Baba 2006
