# Supplementary material for: Structure of the MlaC-MlaD complex reveals molecular basis of periplasmic phospholipid transport
Source: Nat Commun. 2024 Jul 30;15:6394. doi: 10.1038/s41467-024-50615-3 (PMC11289387; doi:10.1038/s41467-024-50615-3)
Supplement: Supplementary file 3 — Description of Additional Supplementary Files [file 41467_2024_50615_MOESM3_ESM.pdf]

<u>Description of Additional Supplementary Files</u>

Supplementary Video 1 – Video of coarsegrained MD simulation showing MlaCD (1:6) (maroon:grey) within a modelled bilayer (green). A lipid of interest (purple) is shown as a ball and stick model. Free lipids (yellow) are present in the experimental frame and can be seen interacting with MlaCD.

Supplementary Video 2 – Video of coarsegrained MD simulation showing MlaCD (2:6) (maroon/blue:grey) within a modelled bilayer (green). Free lipids (yellow) are present in the experimental frame and can be seen interacting with MlaCD.

Supplementary Video 3 – Video of coarsegrained MD simulation showing MlaD (grey) within a modelled bilayer (green). Lipids from the modelled bilayer have moved into the central MlaD pore.
