# Supplementary material for: Structure of the MlaC-MlaD complex reveals molecular basis of periplasmic phospholipid transport
Source: Nat Commun. 2024 Jul 30;15:6394. doi: 10.1038/s41467-024-50615-3 (PMC11289387; doi:10.1038/s41467-024-50615-3)
Supplement: Supplementary file 7 — Reporting Summary [file 41467_2024_50615_MOESM7_ESM.pdf]

nature portfolio

Corresponding author(s): Timothy John Knowles

Last updated by author(s): 14/06/2024

Reporting Summary

Nature Portfolio wishes to improve the reproducibility of the work that we publish. This form provides structure for consistency and transparency in reporting. For further information on Nature Portfolio policies, see our Editorial Policies and the Editorial Policy Checklist.

Statistics

For all statistical analyses, confirm that the following items are present in the figure legend, table legend, main text, or Methods section.

n/a	Confirmed	
[ ]	[x]	The exact sample size (*n*) for each experimental group/condition, given as a discrete number and unit of measurement
[ ]	[x]	A statement on whether measurements were taken from distinct samples or whether the same sample was measured repeatedly
[x]	[ ]	The statistical test(s) used AND whether they are one- or two-sided<br>*Only common tests should be described solely by name; describe more complex techniques in the Methods section.*
[x]	[ ]	A description of all covariates tested
[x]	[ ]	A description of any assumptions or corrections, such as tests of normality and adjustment for multiple comparisons
[ ]	[x]	A full description of the statistical parameters including central tendency (e.g. means) or other basic estimates (e.g. regression coefficient) AND variation (e.g. standard deviation) or associated estimates of uncertainty (e.g. confidence intervals)
[x]	[ ]	For null hypothesis testing, the test statistic (e.g. *F*, *t*, *r*) with confidence intervals, effect sizes, degrees of freedom and *P* value noted<br>*Give P values as exact values whenever suitable.*
[x]	[ ]	For Bayesian analysis, information on the choice of priors and Markov chain Monte Carlo settings
[x]	[ ]	For hierarchical and complex designs, identification of the appropriate level for tests and full reporting of outcomes
[x]	[ ]	Estimates of effect sizes (e.g. Cohen's *d*, Pearson's *r*), indicating how they were calculated

Our web collection on statistics for biologists contains articles on many of the points above.

Software and code

Policy information about availability of computer code

Data collection	Unicorn 5 (Cytiva), CryoSparc, i-control (Tecan), RaptorX, Gromacs, INSANE 3.0,
Data analysis	Graphpad prism, ChimeraX, Inkscape, Pymol

For manuscripts utilizing custom algorithms or software that are central to the research but not yet described in published literature, software must be made available to editors and reviewers. We strongly encourage code deposition in a community repository (e.g. GitHub). See the Nature Portfolio guidelines for submitting code & software for further information.

Data

Policy information about availability of data

All manuscripts must include a data availability statement. This statement should provide the following information, where applicable:

- Accession codes, unique identifiers, or web links for publicly available datasets
- A description of any restrictions on data availability
- For clinical datasets or third party data, please ensure that the statement adheres to our policy

The structures and particle stacks produced in this study are available in the following databases:
● Protein structure: Cryo-electron microscopy structures
Protein Data Bank 8OJ4 (1:6) (http://www.rcsb.org/pdb)
Protein Data Bank 8OJG (2:6) (http://www.rcsb.org/pdb)

Electron Microscopy Data Bank EMD-16904 (1:6) (https://www.ebi.ac.uk/emdb)
Electron Microscopy Data Bank EMD-16913 (2:6) (https://www.ebi.ac.uk/emdb)
● Cryo-electron microscopy: Particle stacks
Electron Microscopy Public Image Archive 47485412 (https://www.ebi.ac.uk/empiar)

DATA AVAILABILITY

Source data are provided with this paper. The 1:6 structure of MlaCD generated in this study has been deposited in the PDB database under accession code 8OJ4 [https://www.rcsb.org/structure/unreleased/8OJ4] as well as in the EMDB database under accession code EMD-16904 [https://www.ebi.ac.uk/emdb/EMD-16904]. The 2:6 structure of MlaCD generated in this study has been deposited in the PDB database under accession code 8OJG [https://www.rcsb.org/structure/unreleased/8OJG] as well as in the EMDB database under accession code EMD-16913 [https://www.ebi.ac.uk/emdb/EMD-16913]. The initial and final configuration of all molecular dynamics trajectories generated in this study have been deposited in the Zenodo database as 'Coarse-Grained Molecular Dynamics simulations of Mla' [https://doi.org/10.5281/zenodo.11492165]. The Cryo-EM particle stack generated in this study has been deposited in the Electron Microscopy Public Image Archive under accession code 47485412.

The structure of MlaD32-183 generated by Ekiert et al. (2017) and used in this study is available in the PDB database under accession code 5UW2 [https://doi.org/10.2210/pdb5UW2/pdb]. The structure of MlaC generated by Ekiert et al. (2017) and used in this study is available in the PDB database under accession code 5UWA [https://doi.org/10.2210/pdb5UWA/pdb].

Research involving human participants, their data, or biological material

Policy information about studies with human participants or human data. See also policy information about sex, gender (identity/presentation), and sexual orientation and race, ethnicity and racism.

Reporting on sex and gender	N/A
Reporting on race, ethnicity, or other socially relevant groupings	N/A
Population characteristics	N/A
Recruitment	N/A
Ethics oversight	N/A

Note that full information on the approval of the study protocol must also be provided in the manuscript.

Field-specific reporting

Please select the one below that is the best fit for your research. If you are not sure, read the appropriate sections before making your selection.

☒ Life sciences ☐ Behavioural & social sciences ☐ Ecological, evolutionary & environmental sciences

For a reference copy of the document with all sections, see nature.com/documents/nr-reporting-summary-flat.pdf

Life sciences study design

All studies must disclose on these points even when the disclosure is negative.

Sample size	n=3 technical replicates
Data exclusions	No excluded data
Replication	n=3 technical replicates. All attempts to repeat the experiments were successful
Randomization	N/A
Blinding	N/A

Reporting for specific materials, systems and methods

We require information from authors about some types of materials, experimental systems and methods used in many studies. Here, indicate whether each material, system or method listed is relevant to your study. If you are not sure if a list item applies to your research, read the appropriate section before selecting a response.

Materials & experimental systems

n/a	Involved in the study
☐	☒ Antibodies
☒	☐ Eukaryotic cell lines
☒	☐ Palaeontology and archaeology
☒	☐ Animals and other organisms
☒	☐ Clinical data
☒	☐ Dual use research of concern
☒	☐ Plants

Methods

n/a	Involved in the study
☒	☐ ChIP-seq
☒	☐ Flow cytometry
☒	☐ MRI-based neuroimaging

Antibodies

Antibodies used	Rabbit alpha-MlaC primary antibody was used at a dilution of 1:500 (Pacific Immunology, kindly supplied by Shu-sin Chng). Secondary (HRP) Goat pAb to Rb IgG (Abcam, Lot:GR3307521-1) used at 1:5000 dilution.
Validation	Validated previously by Yeow J, Luo M, Chng SS. Molecular mechanism of phospholipid transport at the bacterial outer membrane interface. Nature communications 14, 8285 (2023).

Plants

Seed stocks	N/A
Novel plant genotypes	N/A
Authentication	N/A
